# Diagnostic Efficacy of Cardiac Scintigraphy with ^99m^Tc-Pyrophosphate for Latent Myocardial Inflammation in Patients with Atrial Fibrillation

**DOI:** 10.1155/2020/5983751

**Published:** 2020-03-16

**Authors:** Julia Ilyushenkova, Svetlana Sazonova, Konstantin Zavadovsky, Roman Batalov, Yuliya Rogovskaya, Yana Anfinogenova, Yurii Lishmanov

**Affiliations:** Cardiology Research Institute, Tomsk National Research Medical Center, Russian Academy of Sciences, 111a Kievskaya Str., Tomsk 634012, Russia

## Abstract

**Objectives:**

This work aimed to study the efficacy of hybrid ^99m^Tc-Pyrophosphate SPECT/CT for diagnosis of latent inflammatory processes in the myocardium of patients with atrial fibrillation (AF).

**Methods:**

The study comprised 34 patients aged 44 ± 9 years with AF of unknown etiology referred for radiofrequency ablation. The data were acquired using hybrid ^99m^Tc-Pyrophosphate SPECT/CT. To evaluate and interpret the results of hybrid study and to determine localization of radiopharmaceutical accumulation, scintigraphic and CT images were fused. SPECT/CT results were compared with data of endomyocardial biopsy.

**Results:**

Sensitivity, specificity, and accuracy of ^99m^Tc-Pyrophosphate SPECT/CT in diagnosing myocarditis were 91%, 100%, and 94%, respectively. Proposed diagnostic criteria for myocarditis comprised intensity of the radiopharmaceutical accumulation in the myocardium and the ratios of focus/lung, focus/vertebral column, and focus/LV pool. Minimum cutoff values for the histologically verified myocarditis were >1.47 for focus/lung index, >0.11 for focus/vertebral column ratio, and >1.26 for focus/lung index.

**Conclusions:**

SPECT/CT-based quantitative assessment of ^99m^Tc-Pyrophosphate accumulation in the myocardium is a highly informative noninvasive method for diagnosis of inflammatory process in the heart in patients with AF of undefined etiology.

## 1. Introduction

Atrial fibrillation (AF) is the most common type of arrhythmia and is a challenging problem of modern medicine. Despite the fact that AF is not a life-threatening heart rhythm disorder, its presence causes a twofold increase in mortality in cardiac patients and increases the risks of sudden cardiac death, ischemic stroke, and heart failure by 1.3, 5, and 3.4 times, respectively [1].

Elucidation of AF etiology is of primary clinical concern and is ultimately required for successful treatment of this pathology. It is known that the main causes of AF comprise arterial hypertension and coronary artery disease (CAD), but, in 10–20% of cases, the cause of this arrhythmia remains unknown [2, 3]. It is assumed that latent myocardial inflammation may be etiologic and pathogenetic factors in idiopathic AF. The available literature presents a large number of studies focusing on the atrial pathomorphology and the levels of immune and biochemical markers demonstrating relationships between inflammation and AF development [4, 5].

Unfortunately, accurate and timely diagnosis of myocardial inflammation especially when it is a chronic condition presents difficulties [6]. It is widely thought that the diagnosis of myocarditis can be verified only based on a histological analysis of endocardial biopsy material [7]. However, this method is invasive and is associated with 9% risk of complications including tricuspid valve insufficiency with an increase in pulmonary artery systolic pressure, cardiac wall perforation, hemopericardium, cardiac tamponade, and heart rhythm and conduction disorders following this procedure [8]. In the context of myocarditis detection, the methods of nuclear medicine may be promising as they allow for noninvasive and specific evaluation of pathophysiological processes occurring in the affected organ. Over the last years, the methods of integrated radionuclide diagnostics of myocarditis have been implemented in clinical practice. These methods are based on the fusion of scintigraphic images obtained with administration of radiopharmaceuticals for inflammation diagnostic (^99m^Tc-HMPAO-autoleukocytes, ^99m^Tc-Pyrophosphate) and the myocardial perfusion images [9]. Indeed, the efficacy of single-photon emission computed tomography (SPECT) with ^99m^Tc-Pyrophosphate has been demonstrated for evaluation of latent inflammatory changes in the heart of patients with persistent form of AF [9, 10]. At the same time, these radionuclide imaging approaches are multistage, require administration of two radiopharmaceuticals, and do not allow for accurate anatomic identification of the foci of indicator accumulation beyond the left ventricular (LV) contours.

Mentioned disadvantages can be avoided via the use of hybrid devices combining the gamma camera and X-ray computed tomography scanner. Nowadays, SPECT/CT equipment is broadly used to diagnose diseases of various organs and systems, in particular, in patients with cardiologic and cardiac surgery profiles [11]. The efficacy of this image fusion method has been demonstrated for visualization of large and high-intensity foci in pathological accumulation of radiopharmaceutical corresponding to the active inflammatory process in cardiovascular structures. At the same time, capabilities of SPECT/CT for diagnosis of chronic or latent inflammation in the heart in the presence of small and moderately intensive foci of the radiopharmaceutical accumulation remain virtually unknown.

The aim of this work was to study the efficacy of hybrid SPECT/CT method with ^99m^Tc-Pyrophosphate (^99m^Tc-PYP) for diagnosis of latent inflammatory processes in the myocardium of patients with AF.

## 2. Materials and Methods

### 2.1. Patient Selection

The study comprised 34 patients aged 44 ± 9 years suffering from AF [12] of unknown etiology referred for radiofrequency ablation. Before intervention, all patients received complete clinical and instrumental examinations and myocardial ^99m^Tc-Pyrophosphate scintigraphy combined with multislice computed tomography (SPECT/CT). During radiofrequency ablation, the endomyocardial samples were obtained in all patients for histological verification of myocarditis. Baseline characteristics of patients are presented in Table 1.

Criteria of inclusion of patients into the study were as follows:

- Age of 18 to 60 years
- DRUG-resistant persistent form of AF of unknown etiology
- LV volume less than 150 mL according to multislice CT
- The absence of intracavitary thrombi and effect of spontaneous contrast according to data of transesophageal ultrasound study
- The absence of significant coronary artery stenosis (≥50%) according to coronary CT angiography
- Written informed consent of patients for participation in the study

Criteria of exclusion of patients from the study were as follows:

- The presence of severe comorbid pathology (systemic diseases, diabetes, arterial hypertension, hyperthyroidism, etc.)
- Valvular heart disease
- Past history of myocardial infarction/acute myocardial infarction
- Coronary artery disease
- Contraindications for contrast-enhanced multislice CT

After enrolment into the study, patients were assigned into two groups based on the results of pathomorphological examination. Group 1 comprised patients with histologically verified myocarditis. Group 2 comprised patients without signs of myocarditis.

All studies were approved by the Ethics Committee and performed in compliance with ethical standards of provided by the Declaration of Helsinki (1964).

### 2.2. Data Acquisition

#### 2.2.1. Cardiac SPECT/CT

**Figure 1:**
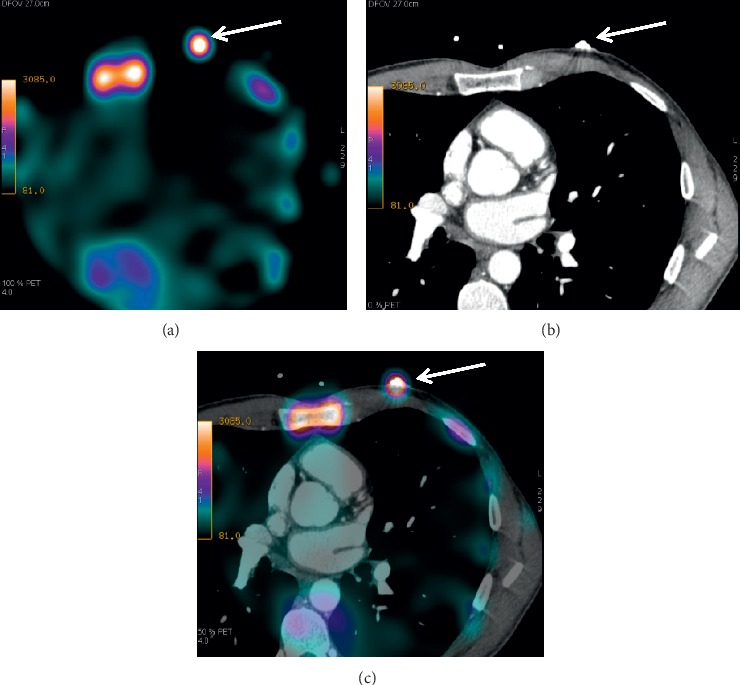
An example of a fusion of the scintigraphic and CT images based on the use of surface markings. (a) Scintigraphic image (axial slice) where arrow shows a position of the radioactive marking; (b) CT tomographic image (axial slice) where arrow shows a position of the radiopaque marking; (c) SPECT/CT image where arrow shows a superposition of two markings.

Cardiac SPECT study was performed 3 and 24 hours after intravenous infusion of 370 MBq of ^99m^Tc-Pyrophosphate [9, 10]. Scintigraphic images were acquired by hybrid SPECT/CT scanner GE Discovery NM/CT 570c (GE Healthcare, Milwaukee, WI, USA) equipped with solid state cadmium-zinc-telluride detectors. Immediately before the scintigraphic study, radionuclide marking was placed on the chest of a patient (over the third intercostal space along the mamillary line). The ECG lead was placed over this radionuclide marking to serve as a radiopaque marking (see Figure 1).

The SPECT data was acquired using low-energy multipinhole collimator and 19 stationary detectors simultaneously imaging 19 different views without detector rotation. Each detector contained 32 × 32 pixelated (2.46 × 2.46 mm) CZT elements. 20% energy window at 140 keV was used. The acquisition time was 400 to 600 s depending on body weight of a patient.

CZT images were reconstructed on the dedicated workstation (Xeleris 4.0; GE Healthcare, Haifa, Israel) using maximum-penalized-likelihood iterative reconstruction (60 iterations; Green OSL Alpha 0.7; Green OSL Beta 0.3) to acquire perfusion images in standard cardiac axes (short axis, vertical long axis, and horizontal long axis). The software Myovation for Alcyone (GE Healthcare, Haifa, Israel) was used for image reconstruction, and Butterworth postprocessing filter (frequency 0.37; order 7) was applied to the reconstructed slices. The reconstruction was performed in 70 × 70 pixels matrix with 50 slices.

**Figure 2:**
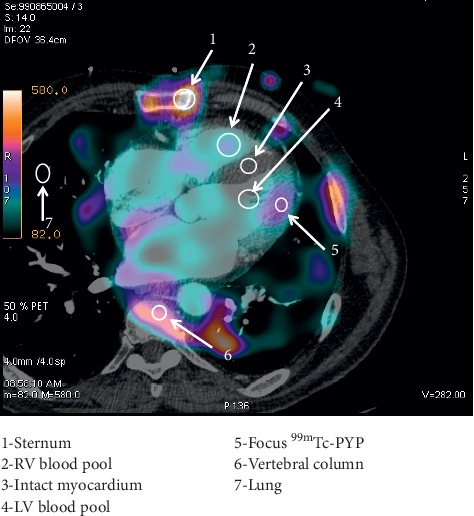
Schematic representation of an approach to an assessment of regions of interest.

The intensities of the radiopharmaceutical accumulations in the regions of interest (ROIs) corresponding to the foci of the elevated radiopharmaceutical accumulation in the myocardium, intact myocardium, lung, sternum, vertebral column, and the LV cavity were quantitatively assessed (see Figure 2). All ROIs were measured at one slice and had equal size of 16 pixels (1 pixel = 4 × 4 × 4 mm); average count was used for further calculations. After that, ratios of the focus/vertebral column, focus/lung, focus/intact myocardium, and focus/LV pool were calculated for subsequent intergroup comparisons.

#### 2.2.2. MDCT Angiography

After completing the SPECT acquisition, multislice CT was performed. Retrospective or prospective (depending on heart rate) ECG-synchronized helical scan (0.18:1–0.24:1 pitch) was used with 120–140 kV tube voltage, 180–700 mAs current, 0.35 s tube rotation, and 0.6 mm slice width.

Contrast enhancement of the coronary arteries, large vessels, and cardiac chambers was performed by intravenous infusion of 70–110 mL (depending on body weight) of iodine-containing contrast agent (iodine concentration of 350–370 mg/mL) at 5 mL/s rate. Obtained data were reconstructed in the diastole phase (mostly, 75% of RR interval duration) and analyzed using Advantage Workstation 4.6, GE Healthcare [13]. Multislice CT angiography was indicated for preoperative examination aimed at evaluation of pulmonary vein ostia sizes and anatomy; assessment of frontal, sagittal, and transverse sizes of the left atrium and its volume; volumetric reconstruction of the left atrium; and ruling out obstructive coronary artery disease [14].

Extent of atherosclerosis in coronary arteries was evaluated based on 17-segment model of the American Heart Association [15]. Stenotic narrowing was considered obstructive if arterial diameter was decreased by ≥50%. Besides coronary artery examinations, the cardiac valves, myocardial structure, and the cardiac chambers sizes were studied.

#### 2.2.3. Fusion Technique and Hybrid Images Analysis

To evaluate and to interpret the results of hybrid study and to determine localization of the radiopharmaceutical accumulation, the scintigraphic and CT images were fused by precise superimposition of the radionuclide and radiopaque markings at the frontal, sagittal, and transverse slices by using Advantage Workstation 4.6 with Fusion QC software application (GE Healthcare).

At the first stage, the acquired tomographic slices were evaluated visually. Foci of the radiopharmaceutical accumulation were considered positive if the areas of ^99m^Tc-Pyrophosphate were located over the myocardium and if the intensity of these foci visually exceeded that of the background. Accumulation of the radiopharmaceutical was considered focal if it was detected within one myocardial segment (according to the 17-segment model of the left ventricle [16]) and it was considered diffuse if it was detected in two or more segments. At the second stage of image analysis, intensity of the radiopharmaceutical accumulation was calculated for various structure of the mediastinum via the automatic impulse counting in the ROIs with a following determination of focus/lung, focus/vertebral column, focus/sternum, focus/lung, focus/intact myocardium, and focus/LV blood pool coefficients.

All images were reevaluated independently by two experienced nuclear medicine physicians who were not aware of the patients' clinical history and of the results of prior conventional imaging.

#### 2.2.4. Endomyocardial Biopsy

Histology material was sampled during radiofrequency ablation of the ectopic foci. Catheterization of the right heart was performed through the fluoroscopy-guided transvenous approach by the method proposed by Saldinger. The fragments of biopsy material were sampled from the apex of the right ventricle (one fragment), outflow tract of the right ventricle (two fragments), and the interventricular septum on the right ventricular side (two fragments) by using Biopsy Forceps 7F, 50/100 cm (Cordis, USA). To detect histologic signs of myocarditis, we used Dallas criteria [17] and classification of myocarditis proposed by Calabrese et al. [18].

The SPECT/CT results were compared with data of endomyocardial biopsy.

#### 2.2.5. Statistical Processing of Data

Statistical processing of data was performed with SPSS 20 software. The distribution of continuous variables was determined by Shapiro–Wilk W test. Therefore, Mann–Whitney *U* test was used. The values of sensitivity, specificity, and diagnostic accuracy, as well as the positive and negative predictive values of the method, were calculated based on generally accepted formulas and ROC curves. Interobserver variability was calculated using multirater Cohen's kappa (*κ*) statistics with a 95% confidence interval (CI). The definitions presented by Landis and Koch were used to evaluate the strength of the rater agreement and were categorized as slight (0–0.20), fair (0.21–0.40), moderate (0.41–0.60), substantial (0.61–0.80), and almost perfect (0.81–1.00). A 2-tailed *p* value <0.05 was considered significant [19].

## 3. Results

### 3.1. Patient Characteristics

At time of admission, 24 patients (70%) presented with complaints of cardiac rhythm disorder. Patients experienced episodes of heart palpitations in 47% of cases (*n* = 11), skipped heart beats in 30% of cases (*n* = 7), and constant heart racing in 13% of cases (*n* = 6). Four patients (12%) complained of discomfort in the cardiac region; 13 patients (38%) complained of shortness of breath during moderate physical activity. Ten patients (29%) did not have any complaints, and AF was detected as an incidental finding during the annual preventive medical examination (see Table 1).

Laboratory examinations did not show any signs of inflammatory process in patients.

According to data of 24-hour Holter ECG monitoring, episodes of AF were registered in all patients and were associated with single premature ventricular contractions in 100% of cases.

According to echocardiography study, the values of ejection fraction, end-diastolic volume, end-systolic volume, and contractile function of the left ventricle were within normal ranges in all patients.

### 3.2. Histology Examination Results

Based on the results of histologic study of the right ventricular endomyocardial biopsy, myocarditis was verified in 24 (70.5%) out of 34 patients with AF. Based on cellular composition of the infiltrate, myocarditis was classified as lymphocytic in 21 patients (61%) and as polymorphcellular in three patients (8.8%).

In regard to of lesion extent, 18 patients (75%) had diffuse myocarditis, 2 patients (8.3%) had focal myocarditis, and 4 patients (16.6%) had the resolution stage of myocarditis. Along with cellular infiltration, the areas of fibrosis were found in all patients.

Ten patients (29.5%) had verified fibrosis varying in its extent between patients. Interstitial fibrosis was observed in 6 out of 10 patients. Mild fibrosis affecting the area less than 20% was detected in 2 patients, moderate fibrosis affecting 20% to 40% area was found in 4 patients, and severe fibrosis with area of over 40% was detected in one case.

### 3.3. SPECT/CT Results

Multislice MDCT angiography data did not show any signs of coronary atherosclerosis, valvular disease, myocardial structure abnormalities, and increases in ventricular volumes in patients. The left atrial volume exceeded 90 cm^3^ in 14 patients (41%).

At the scintigraphic images acquired 3 hours after the administration of the radiopharmaceutical, there was an intensive visualization of intraventricular blood pool, which hindered a visual analysis of the images and could affect confidence of ROI values. In this context, the scintigrams acquired 24 hours after the ^99m^Tc-Pyrophosphate infusion were used for the analysis. At the delayed images, blood clearance was sufficient for the adequate analysis.

**Figure 3:**
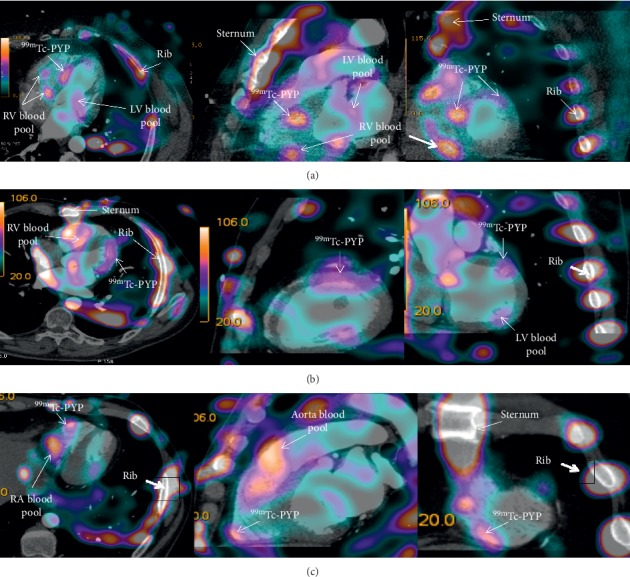
^99m^Tc-Pyrophosphate-based SPECT combined with multislice CT angiography. (a) Arrows show high-intensity focal accumulation of the radiopharmaceutical in the middle septal part of the left ventricle and low-intensity accumulation in the middle septal part and the anterolateral region of the left ventricle. (b) Arrows show diffuse moderate-intensity pathological accumulation of ^99m^Tc-Pyrophosphate in the middle basal part of the left ventricle. (c) Arrows show focal pathological accumulation of ^99m^Tc-Pyrophosphate in the middle part of the right ventricular free wall.

The foci of ^99m^Tc-Pyrophosphate uptake in the LV myocardium were observed in 22 out of 34 patients (73.5%) of whom 3 patients (13.6%) had accumulation of the radiopharmaceutical in the right ventricular myocardium (see Figure 3). The pattern of the radiopharmaceutical accumulation was focal in 20 patients (90%) and diffuse in 2 patients (9%). The radiopharmaceutical accumulation in one wall of the left ventricle was observed in 10 patients (45.5%) and in two and more walls in 12 patients (54.5%). The more detailed characteristics of the ^99m^Tc-Pyrophosphate accumulation are presented in Table 2.

Significant intergroup differences in all calculated coefficients were observed (see Table 3). The greatest differences were found in the coefficients of focus/lung (by 41%), focus/intact myocardium (by 35%), focus/LV blood pool (by 28%), focus/sternum (by 25%), and focus/vertebral column (by 20%).

Interobserver agreement between the 2 measurement ROIs by 2 observers was almost perfect for all values: sternum *κ* = 0.907 (CL 95% 0.869–0.945; *p* < 0.001), lung *κ* = 0.862 (CL 95% 0.821–0.904; *p* < 0.001), vertebral column *κ* = 0.942 (CL 95% 0.922–0.963; *p* < 0.001), LV blood pool *κ* = 0.874 (CL 95% 0.830–0.917; *p* < 0.001), intact myocardium *κ* = 0.845 (CL 95% 0.779–0.910; *p* < 0.001), and focus *κ* = 0.904 (CL 95% 0.877–0.931; *p* < 0.001).

**Figure 4:**
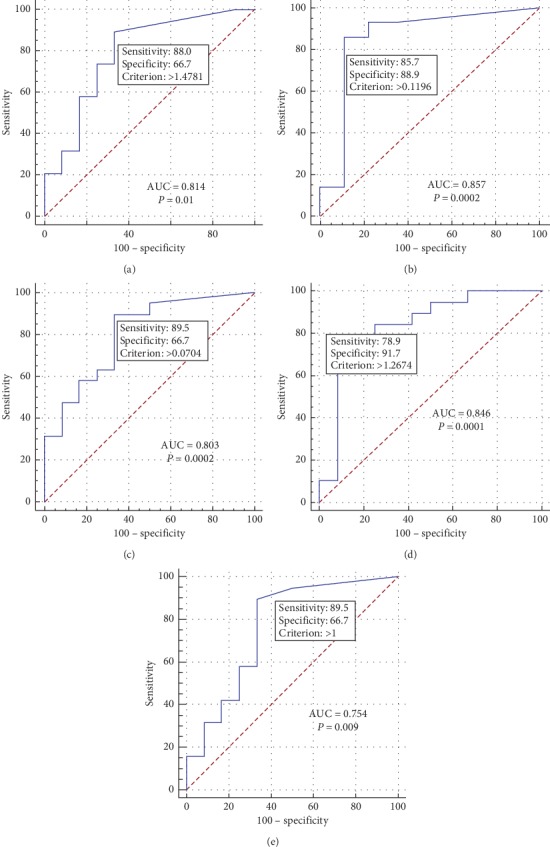
ROC curves for diagnostic values of different coefficients in the diagnosis of myocarditis in patients with atrial fibrillation. (a) ROC curve for the coefficient of focus/lung; (b) ROC curve for the coefficient of focus/vertebral column; (с) ROC curve for the coefficient of focus/sternum; (d) ROC curve for the coefficient of focus/LV pool; and (e) ROC curve for the coefficient of focus/intact myocardium.

Based on the ROC analysis, the coefficients of focus/vertebral column, focus/LV pool, and focus/lung had the highest sensitivity and specificity in diagnosis of myocarditis (see Figure 4, Table 4).

**Figure 5:**
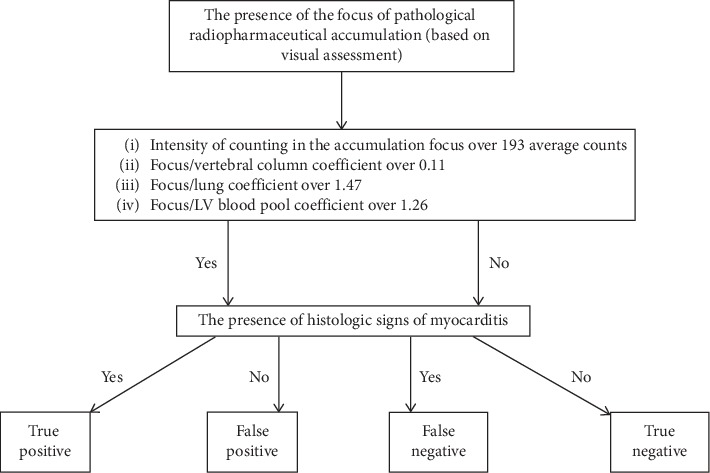
Schematic representation of the attribution of results to true and false groups when the combination of the visual and quantitative assessments was used for the diagnosis of myocarditis.

In the case of combination of visual and quantitative scintigraphic image assessments (see Figure 5), the numbers of true-positive, true-negative, false-positive, and false-negative results were 22, 10, 0, and 2, respectively. The values of sensitivity, specificity, and accuracy for SPECT/CT with ^99m^Tc-Pyrophosphate were 91%, 100%, and 94%, respectively. The positive predictive value of the method was 100% and negative predictive value was 83%.

## 4. Discussion

The main finding of the study is that the visual and semiquantitative analysis of merged SPECT and CT images allowed diagnose myocarditis, confirmed by endomyocardial biopsy, with the high level of sensitivity, specificity, and accuracy—91%, 100%, and 94%, respectively.

Several studies have shown that inflammatory processes in the myocardium play a pivotal role as an etiologic factor along with arterial hypertension and coronary artery disease in the development and maintaining of AF [20–22]. According to data of various authors, histological signs of inflammation in patients with AF of unspecified etiology are present in 14–70% of cases [21–23]. Unfortunately, only endomyocardial biopsy allows verifying the diagnosis of myocarditis. However, considering invasiveness of this procedure, endomyocardial biopsy is taken only in limited population of patients and is inadvisable in patients with AF [24]. Meanwhile, the state-of-the-art detection of the signs of myocardial inflammation may affect strategy of treatment for AF in a framework of upstream therapy [25].

The development of the state-of-the-art nuclear medicine technologies offers new opportunities on the way of solving problems of diagnosing inflammatory diseases of cardiovascular system. In the previous works, we used dual-isotope method of study, namely, SPECT with ^99m^Tc-PYP and perfusion SPECT with ^99m^Tc-MIBI. The diagnostic strength of that approach was lower and the sensitivity, specificity, and accuracy values were 80%, 83%, and 82%, respectively [10]. Besides, a capability to visualize all cardiac chambers was unavailable. In the present work, we used the fusion of scintigraphic images and computed tomographic angiography which provided opportunity to reach relatively high values of diagnostic efficiency of visualization of focal inflammatory changes and to decrease the time of patient examination.

Although ^99m^Tc-PYP is inherently a marker of myocardial injury (myocardial infarction), its use is also reasonable for detection of myocarditis because inflammation is accompanied by the alternative processes. However, considering the recommendations for most radiopharmaceuticals used to diagnose inflammation, we increased the time interval between the ^99m^Tc-PYP injection and image acquisition recommended by protocols (1 to 3 h) to 24 hours (delayed image acquisition) [26]. It allowed getting rid of the intracardiac and intravascular pools and decreasing the number of false-positive results as much as possible. We have used this approach since 2013 [9, 10] and it was approved using the large number of patients and proved to be valuable.

In regard to lesion extent, the pattern of the ^99m^Tc-PYP uptake in the left ventricle was focal in 20 patients (90%) and diffuse in two patients (9%). However, 18 patients (75%) had diffuse myocarditis based on the results of histologic study of the RV biopsy. This paradox may be due to the fact that the sizes of ^99m^Tc-PYP accumulation corresponded to the area of myocardial injury, but not the area of cellular infiltration within it (according to Duke criteria). Therefore, a scintigraphic pattern of the focal and diffuse accumulations of the radiopharmaceutical may differ from the histologic pattern.

According to the literature, the standard for estimation of radiopharmaceutical uptake in the myocardium is heart/mediastinal blood pool ratio, especially when studies with 123I-MIBG are performed [27]. However, the calculation of the heart/mediastinum blood pool ratio when using ^99m^Tc-PYP is complicated by an intensive uptake of the radiopharmaceutical in the sternum. For scintigraphic diagnostics of cardiac amyloidosis using ^99m^Tc-PYP, H/C index (heart/contralateral zone) on planar and tomographic images is calculated [19, 28–30]. The value of heart/contralateral zone index ranges within 1.5–1.7 [31, 32] and is also used for prediction of patient survival [28]. It is of importance that, in the majority of cases, indices are calculated by using planar images. Moreover, a few works were carried out using other specific and nonspecific indicators of inflammation based on visual and semiquantitative assessment of accumulation proposed by Parkey et al. [33, 34]. At the same time, there are no current recommendations for SPECT ^99m^Tc-PYP scintigraphy analysis for diagnosis of myocarditis. In our opinion, it is difficult to use H/C coefficient (heart/contralateral zone) to assess the degree of ^99m^Tc-PYP uptake in the myocardium at myocarditis, because the inflammatory foci on the images are quite small and radioactivity from soft tissue structures and the blood pool of blood vessels can significantly affect the count of impulses in the ROI drawn from the whole myocardium. Therefore, the objects of our research interest were several coefficients calculated on SPECT images, particularly focus/left ventricular blood pool ratio, which showed high values of diagnostic efficiency.

Capability of visualization of the radiopharmaceutical accumulation foci in the right ventricle is questionable. The histologic material was harvested from the right ventricle in all patients and myocarditis was verified only in 24 patients. However, the focal pathological accumulation of ^99m^Tc-Pyrophosphate was detected only in three patients because there were serious challenges for adequate assessment of the radiopharmaceutical uptake in the right ventricular myocardium due to the artifacts from the bone structures of the chest caused by the pattern of ^99m^Tc-Pyrophosphate accumulation. Solution of this problem, in our opinion, may consist in the use of a specific indicator which cannot accumulate in bone structures of the chest. Nevertheless, there are no available literature data regarding visualization of the inflammation in the right ventricle, which perhaps may be explained by the fact that inflammatory process in the right ventricle does not have much significance for the treatment of this category of patients.

We did not perform visual and semiquantitative assessments of the radiopharmaceutical accumulation in the left atrial walls because radioactive pool of blood in the left atrial cavity could be easily confused with the focal pathological accumulations, whereas histological sampling in this zone for comparative evaluation was not feasible. Nevertheless, Kotani et al. [35] reported feasibility of visualization of the atrial myocardium based on SPECT/CT with Gallium-67. Authors reported that the use of this method was necessary to clarify localizations of radiopharmaceutical accumulation in cases when the accumulation was present on the planar images.

Therefore, SPECT/CT-based semiquantitative assessment of the ^99m^Tc-Pyrophosphate accumulation in the myocardium presents a highly informative and noninvasive method for the diagnosis of inflammatory process in the heart in patients with AF of undefined etiology.

## 5. Study Limitation

The limitation of this study is a small size of study population. Besides, no histological specimens from the LV were obtained in patients included in the study. While planning this work, we complied with the 2007 AHA/ACCF/ESC Scientific Statement, “The role of endomyocardial biopsy in the management of cardiovascular disease” [24]. According to this statement, performing EMB is not recommended in patients with atrial fibrillation unknown etiology (class of recommendation III, level of evidence C). Moreover, EMB from the RV is the safest in terms of the development of complications. At the same time, we relied on the works of Frustaci et al. [23] suggesting the presence of inflammatory process in the ventricles and atria of the heart.

Another limitation of the study is the impossibility of completely eliminating spatial mismatch of the SPECT/CT images. This mismatch occurs due to the respiratory motion of the chest, natural motions of the heart and large vessels, and involuntary muscle contractions that cannot be completely eliminated without using the additional methods of correction. Livieratos [36] provides several methodological approaches to minimize the mismatch. Among them are the correction of patient's motion, the correction of heart and large vessels motion, and the correction of artifacts associated with breathing. In the present work, we used labels to correct the patient's motion, which later allowed us to precisely combine images in three planes. A promising approach to reduce cardiac motion artefacts is to use the three-dimensional nonrigid morphing of the nondiastolic bins to the end-diastolic bin prior to bin summation [37]. Correction of the artifacts associated with breathing would be the most promising approach, but it required using the respiratory gating device unavailable in our department. Overcoming these limitations requires further studies.

## 6. Conclusions

^99m^Tc-Pyrophosphate-based SPECT/CT with visual and quantitative assessments is highly sensitive, specific, and accurate (91%, 100%, and 94%, respectively) in diagnosis of myocarditis. Criteria for diagnosis of myocarditis are coefficients focus/lung, focus/vertebral column, and focus/LV pool indices. Minimum cutoff values of the coefficients for diagnosis of myocarditis are >1.47 for focus/lung index, >0.11 for focus/vertebral column ratio, and >1.26 for focus/LV blood pool index.

## Figures and Tables

**Table 1:** Data of clinical and instrumental examinations.

Clinical characteristics	Total (*n* = 34)
Age, years	44 ± 9
Gender	
Men	27
Women	7
Duration of disease, years	5.1 ± 3
Number of paroxysms per year, *M* ± SD	217.7 ± 160
Cardialgia, *n*	4 (12%)
Exertional dyspnoea, *n*	13 (38%)
Episodes of blood pressure elevated over 140/90 mmHg, *n*	4 (12%)
Low-grade fever, *n*	0
Complaint-free, *n*	10 (29%)
Association of AF onset with infection disease, *n*	8 (23%)
Leukocyte count, (10^9^/L), *M* ± SD	5.56 ± 1.22
ESR, mm/h	5.43 ± 4.5
NYHA	
Class 0	30 (88%)
Class I	4 (12%)
Ejection fraction (%), *M* ± SD	63.7 ± 10.4
LV EDV (mL), *M* ± SD	118.2 ± 35.3
LV ESV (mL), *M* ± SD	45.2 ± 32.8
Left atrial volume, (mL), *M* ± SD	113.5 ± 37.5

AF: atrial fibrillation; ESR: erythrocyte sedimentation rate; FC: functional class; LV: left ventricle; EDV: end-diastolic volume; ESV: end-systolic volume.

**Table 2:** Characteristics of the ^99m^Tc-Pyrophosphate accumulation in the walls of the left and right ventricles.

	Localization of the ^99m^Tc-Pyrophosphate accumulation	Number of cases (%)
Single focus	Septum	8/36
	Lateral wall	1/4.5
	Anterior wall	1/4.5

Multiple foci	Septum + lateral wall	4/18
	Septum + posterior wall	2/9
	Septum + anterior wall	3/13.6
	Septum + free right ventricular wall	3/13.6

**Table 3:** Intergroup comparisons of different coefficients.

Coefficient	Myocarditis negative^*∗*^ (*n* = 10) *M* ± SD	Myocarditis positive (*n* = 22) *M* ± SD	Mann–Whitney *U* test
Focus/vertebral column	0.08 ± 0.01	0.4 ± 0.2	*p*=0.004
Focus/sternum	0.1 ± 0.1	0.4 ± 0.3	*p*=0.005
Focus/lung	4.32 ± 5.02	10.56 ± 6.12	*p*=0.002
Focus/intact myocardium	3.82 ± 1.87	10.64 ± 5.8	*p*=0.018
Focus/LV blood pool	1.04 ± 0.7	3.68 ± 2.4	*p*=0.003

LV: left ventricle; RV: right ventricle. ^*∗*^The coefficients were calculated based on a ratio relative to intact myocardium.

**Table 4:** ROC-analysis results.

Coefficient	ROC-analysis data	Sensitivity and specificity, %
Focus/vertebral column	AUC = 0.857 Cutoff value > 0.11 *p*-level = 0.0002	Se = 85.7 Sp = 88.9

Focus/sternum	AUC = 0.803 Cutoff value > 0.07 *p*-level = 0.0002	Se = 89.5 Sp = 66.7

Focus/lung	AUC = 0.814 Cutoff value > 1.47 *p*-level = 0.01	Se = 88 Sp = 66.7

Focus/intact myocardium	AUC = 0.754 Cutoff value > 1 *p*-level = 0.009	Se = 89.5 Sp = 66.7

Focus/LV blood pool	AUC = 0.846 Cutoff value > 1.26 *p*-level = 0.0001	Se = 78.9 Sp = 91.7

AUC: area under the curve; LV: left ventricle; ROC: receiver operating characteristic; Se: sensitivity; Sp: specificity.

## Data Availability

All the initial data are the property of Cardiology Research Institute, Tomsk National Research Medical Center, Russian Academy of Sciences.
